# Feasibility of deep learning-based image reconstruction using TrueFidelity for coronary calcium scoring

**DOI:** 10.1371/journal.pone.0358342

**Published:** 2026-09-18

**Authors:** Ji Won Kim, Tae Hoon Kim, Seung Hyun Lee, Ji Eun Nam, Chul Hwan Park

**Affiliations:** 1 Department of Radiology and the Research Institute of Radiological Science, Gangnam Severance Hospital, Yonsei University College of Medicine, Seoul, Republic of Korea; 2 Department of Integrative Medicine, Yonsei University College of Medicine, Seoul, Republic of Korea; Stamford Health System: Stamford Hospital, UNITED STATES OF AMERICA

## Abstract

We evaluated the feasibility of deep learning-based image reconstruction (DLIR) using GE TrueFidelity at high strength for coronary calcium scoring, compared with filtered back projection (FBP) and iterative reconstruction (IR; adaptive statistical iterative reconstruction-V with 80% blending), using a cardiac phantom and clinical data. Calcium scores for FBP, IR, and DLIR were obtained with a cardiac phantom. Subsequently, 113 patients who underwent coronary computed tomography angiography were retrospectively evaluated. The Agatston method was applied to all three techniques to obtain the coronary artery calcium score (CACS). Differences, correlations, and concordance in sectional classification of calcium scores were evaluated across reconstruction methods. In the phantom study, calcium scores were comparable across reconstruction methods. In the clinical study, DLIR had significantly lower scores than FBP (p < 0.001), whereas the comparison between IR and DLIR was statistically non-significant (p = 0.064). Weighted kappa coefficients indicated high agreement in total CACS classification across the reconstruction methods. However, Bland–Altman analysis demonstrated non-negligible individual-level disagreement between FBP and DLIR, with a mean difference of 14.5 and 95% limits of agreement from −52.1 to 81.1. This disagreement may be clinically relevant near established CAC risk-category thresholds, particularly 0, 100, and 300. Among 113 patients, 109 were classified into the same CAC-DRS category by both FBP and DLIR. DLIR underclassified four patients, yielding a false-negative rate of 4.65%. In conclusion, DLIR was feasible for CACS measurement and showed high overall concordance with FBP and IR. However, near-perfect correlation should not be interpreted as interchangeability, because DLIR showed systematic underestimation and non-negligible individual-level disagreement from FBP, particularly in patients with low calcium burden or scores near CAC risk-category thresholds.

## Introduction

Ischemic heart disease (IHD) remains a leading cause of morbidity and mortality worldwide [1–3]. Given its substantial global burden, considerable efforts have been made to improve risk prediction. Quantifying calcium in the coronary arteries has emerged as a feasible method for predicting IHD [4]. In 1990, Agatston introduced a method for quantifying coronary artery calcium (CAC), now known as the Agatston score, which has become a widely used tool for cardiovascular risk prediction [5–9]. Because filtered back projection (FBP) was the standard reconstruction method at that time, CAC quantification using FBP remains the conventional reference for coronary calcium scoring.

Advances in medical imaging have led to reconstruction techniques such as iterative reconstruction (IR) and deep learning-based image reconstruction (DLIR). More broadly, recent advances in deep learning methods, including physics-informed neural network approaches, have contributed to the development of data-driven image-processing and reconstruction techniques [10]. In the past, IR was not used clinically because of limitations in computer performance. However, advances in computing have enabled the clinical use of IR. Compared with FBP, IR offers noise reduction and lower patient radiation exposure [11,12]. DLIR, developed through advances in artificial intelligence (AI), is a recent addition to medical imaging. Prior studies have shown that DLIR can maintain image quality under reduced-dose acquisition settings [13,14]. However, in the present study, FBP, IR, and DLIR were reconstructed from the same raw acquisition data, and radiation dose was therefore not directly compared across reconstruction methods.

Accordingly, the present study focused on the feasibility of CAC scoring and the potential impact of DLIR on calcium score estimation rather than on dose reduction. Although previous studies have compared IR and DLIR for coronary calcium scoring, the clinical implications of DLIR-related underestimation remain incompletely understood, particularly in patients with low calcium burden [15,16]. Recent studies have reported DLIR-related underestimation of Agatston scores, particularly at higher strength levels [17]. In particular, the clinical relevance of false-negative classification at the CAC 0 threshold remains insufficiently characterized. Therefore, this study aimed to evaluate the feasibility of using DLIR for CAC scoring by comparing results with those obtained from FBP and IR using a semiautomated coronary calcium scoring workflow, with particular attention to underestimation and false-negative classification.

## Methods

### Ethics statement

This retrospective study was approved by the ethics committee and institutional review board of Gangnam Severance Hospital (3-2022-0291). The requirement for written informed consent was waived due to the study’s retrospective design. All methods were conducted in accordance with the relevant guidelines and regulations.

### Phantom study

#### Experimental design.

CAC images were acquired using a cardiac phantom (Dynamic Cardiac CT Phantom MD-CT, PH-6B; Kyoto KAGAKU, Kyoto, Japan) at a heart rate of 60 beats/min and an ejection fraction of 50%. The heart phantom is composed of polyurethane and hydroxyapatite (approximately 40 HU). The calcification used in this study was calcium chloride, with a density of 2.15 g/mL and a circumferential shape. The maximum diameter of the calcification was 5 mm, and its volume was calculated as 65.42 mm³. Each measurement was repeated five times under consistent conditions to ensure reproducibility of the phantom image measurements. The parameters for calcium-scoring CT acquired with a 16-cm axial scan were as follows: tube voltage, 120 kVp; tube current, 50 mAs; tube rotation time, 0.28 s; and slice thickness, 2.5 mm.

#### Image reconstruction and calcium scoring.

Phantom images were reconstructed using standard FBP, adaptive statistical IR (ASIR-V, 80% blending; GE Healthcare, Waukesha, WI, USA), and DLIR (TrueFidelity, High strength; TF; GE Healthcare). The reconstructed CAC images were transmitted to commercially available software (Aquarius iNtuition Ver. 4.4.12; TeraRecon, Foster City, CA, USA) for calcium score evaluation. Using this software, the calcium score was calculated semiautomatically using the Agatston method.

### Clinical study

#### Study population.

**Fig 1:**
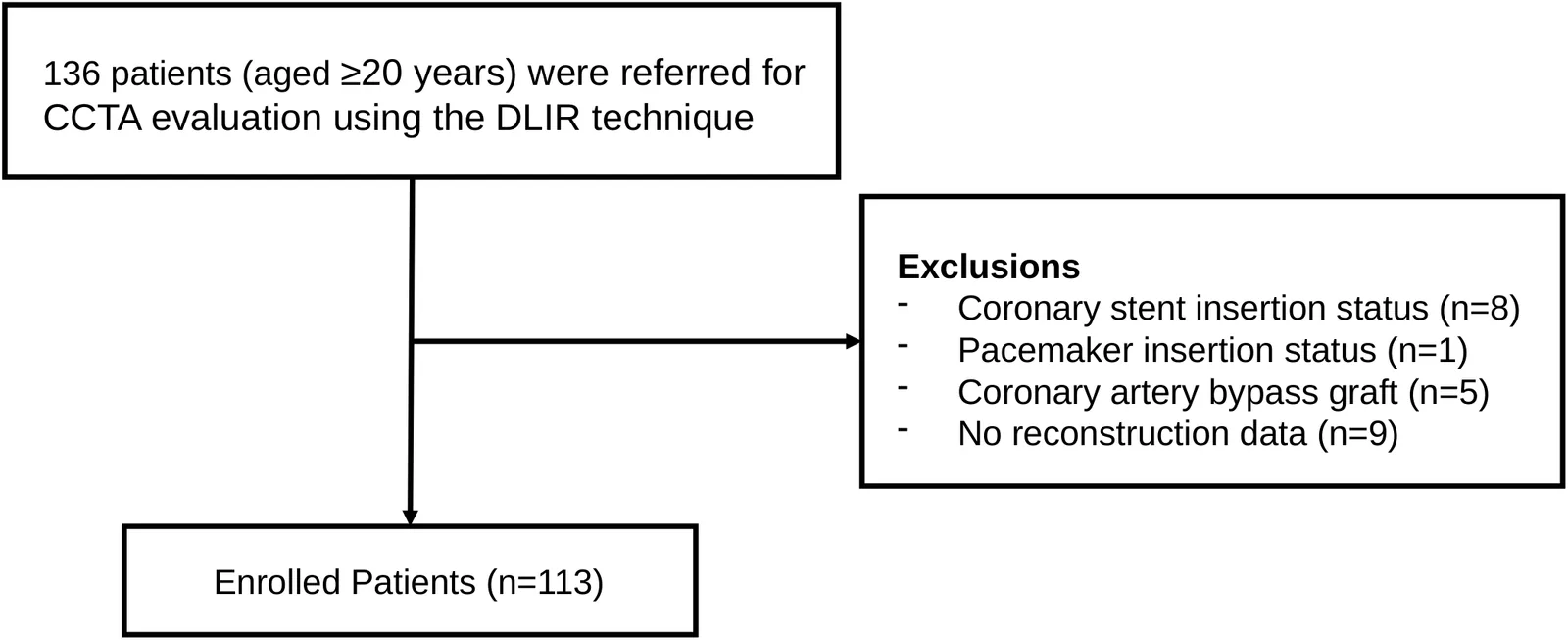
Flow chart of patient selection.

A total of 136 consecutive adult patients who underwent clinically indicated coronary computed tomography angiography (CCTA) and had DLIR reconstruction data available during the study period were initially considered for inclusion. Patients younger than 20 years were not included because coronary artery calcification is uncommon in this age group, making calcium scoring clinically less meaningful. Of these, 23 were excluded for a history of coronary stent insertion (n = 8), pacemaker implantation (n = 1), coronary artery bypass graft surgery (n = 5), or lack of reconstruction data (n = 9). After applying these criteria, 113 patients were ultimately included in the study (Fig 1).

#### CT protocols.

All scans were performed using a 256-slice CT scanner (Revolution CT; GE Healthcare). Calcium scores on CT images for all patients were obtained by specifying a scanning range from the subcarinal level to the base of the heart. CT acquisition parameters are summarized in Table 1.

**Table 1:** Protocols for calcium scoring computed tomography.

	Calcium-scoring CT
Tube voltage, kVp	120
Tube currents, mAs	50
Slice thickness, mm	2.5
Coverage length, cm	16
Rotation time, s	0.28
Scan mode	Axial
Kernel	Standard
Electrocardiogram gating	Prospective triggering
R-R interval	75%

### CT image reconstruction and analysis

CAC images were reconstructed using standard FBP, IR (ASIR-V, 80% blending), and DLIR (TrueFidelity, High strength). All three reconstruction methods were generated from the same raw acquisition data for each patient. Calcium scores were calculated separately from each reconstructed image set using the same threshold-based Agatston scoring method, without sharing segmentation masks or intermediate outputs between reconstruction methods. Data were transferred via a picture archiving and communication system (Centricity 4.0; GE Medical Systems, Mount Prospect, IL, USA) to commercial software (Aquarius iNtuition Ver. 4.4.12; TeraRecon) for CAC measurement. Calcium scoring was performed using a semiautomated workflow in Aquarius software, in which regions exceeding 130 HU were automatically highlighted and then reviewed slice by slice by the reader for confirmation as calcification. Formal blinding to reconstruction method was not performed, and the three reconstruction datasets for each patient were reviewed sequentially. Attenuation (HU) and noise were measured by placing a circular region of interest (ROI) on a cross-sectional image of the ascending aorta between the right and left main coronary arteries. The largest possible ROI was selected to carefully exclude the aortic wall and maintain measurement accuracy. Image noise was defined as the standard deviation (SD) of CT density in the ascending aorta. The SD of attenuation values was used to compare image quality [18]. In the Aquarius software, areas with HU values >130 are colored yellow. The CAC score (CACS) was obtained by examining the area marked by a yellow slice in the axial image and selecting it as CAC. After this selection, the area (mm^2^) was automatically multiplied by the appropriate weight (1 = 130–199 HU; 2 = 200–299 HU; 3 = 300–399 HU; 4 = ≥ 400 HU). The CACS was determined by a radiologist using the Agatston method. The radiation dose for the calcium scoring scan was based on the volume CT Dose Index (CTDI; CTDI_vol_) and the dose–length product (DLP). The DLP was determined by multiplying the CTDI_vol_ by the length of the irradiated region [19].

### Statistical analysis

Demographic characteristics were examined initially. For continuous variables, normally distributed data are presented as mean ± standard deviation, whereas nonparametric data are reported as median [IQR]. Because CACS data showed a zero-inflated distribution with many zeros, mean ± standard deviation was also reported alongside median [IQR] for clarity in interpretation and visualization. Normality was assessed using the Shapiro-Wilk test, and the CACS variables did not satisfy the assumption of normality. Therefore, the Friedman test was used as the primary analysis. Repeated measures ANOVA was additionally reported as a supplementary analysis to provide interpretive context for the zero-inflated CACS data. Pearson’s correlation coefficients and ICC values were calculated as supplementary descriptive measures, whereas agreement-based analyses were treated as the primary measures of clinical agreement across reconstruction methods. The intraclass correlation coefficient (ICC) was calculated for each pairwise comparison between FBP and IR, between FBP and DLIR, and between IR and DLIR using a two-way mixed-effects model with absolute agreement. Furthermore, to analyze differences in FBP, IR, and DLIR on a categorical basis, the CACS was classified into four risk categories (CACS of 0, total < 100, 100 ≤ total < 300, total ≥ 300) according to the Coronary Artery Calcium Data and Reporting System (CAC-DRS) recommended by the Society of Cardiovascular Computed Tomography [20]. Within each category, frequency tables were compiled for accuracy, misclassification, overestimation, and underestimation. Cohen’s kappa statistic quantified agreement for the categorical assignments. Bland–Altman plots were used to assess agreement in continuous CACS. Bowker’s test of symmetry was used to evaluate pairwise differences in the distribution of reclassified patients between FBP and IR, FBP and DLIR, and IR and DLIR. For post hoc pairwise comparisons, Bonferroni correction was applied, with statistical significance defined as p < 0.0167. Calcium scores were independently assessed by two experienced readers in 34 patients (30% of 113 participants), and inter-observer agreement was evaluated using the ICC. The scoring workflow was semiautomated: Aquarius software automatically highlighted regions exceeding 130 HU, and each reader independently reviewed these candidate regions and confirmed or excluded them as calcifications. Because discrepancies between the two readers were negligible, no formal disagreement resolution process was required. All statistical analyses were performed using IBM SPSS Statistics for Windows (version 25.0; IBM Corp., Armonk, NY, USA) or MedCalc Version 19.6.4 (MedCalc Software, Ostend, Belgium).

## Results

### Phantom study

**Fig 2:**
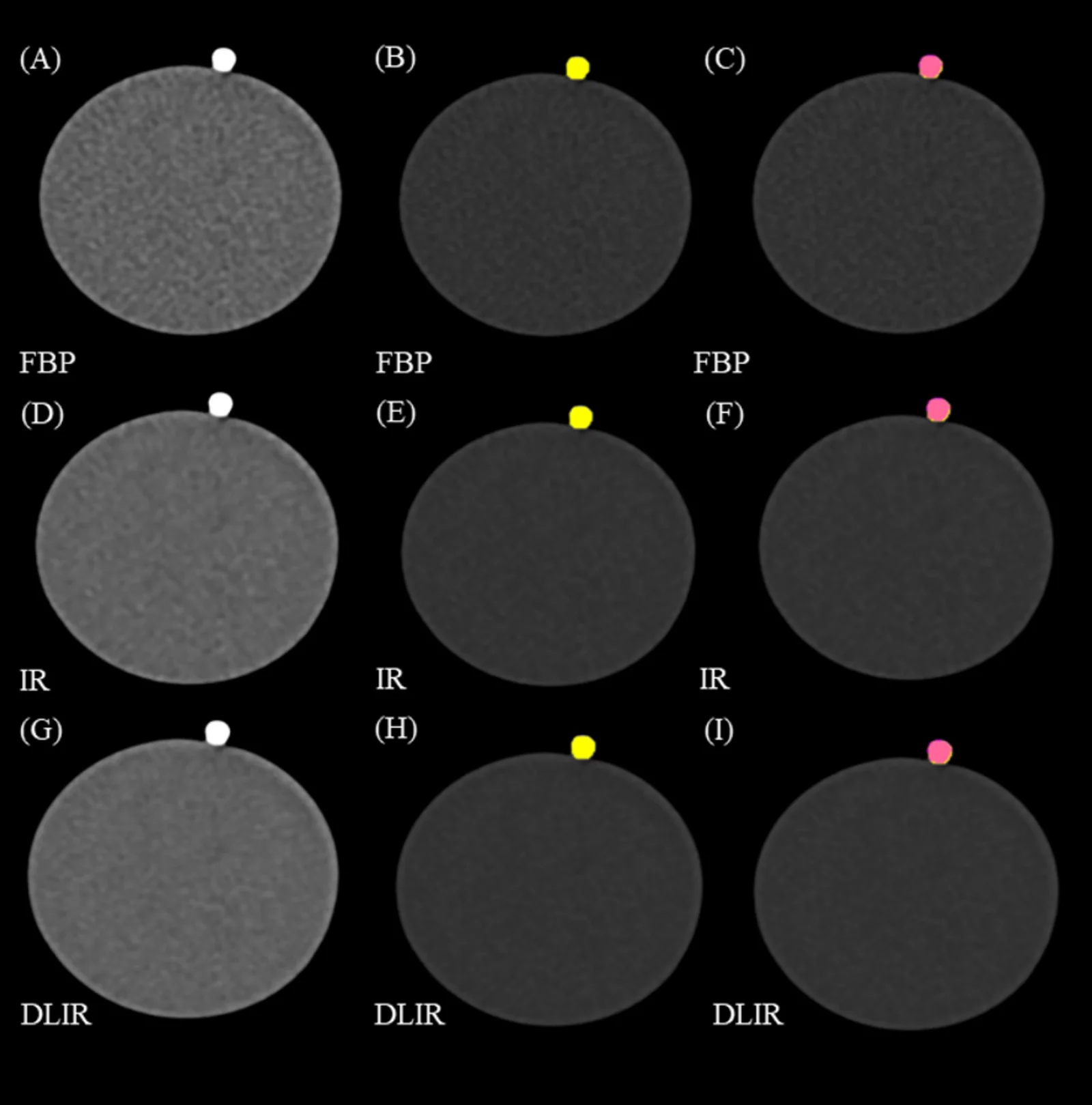
Comparison of calcium scores obtained using FBP, IR, and DLIR in a phantom. A phantom study was conducted using a dynamic cardiac phantom and calcium chloride. Commercially available software displays calcium with ≥130 HU in yellow. After manual selection, calcium is shown in pink, and the calcium score is measured semiautomatically. From left to right, the original image, the image identified as calcium by the software, and the image designated as calcium by the author are shown. The calcium score and image noise for each reconstruction method were as follows: For FBP (A–C), the calcium score was 59.3, and the image noise was 10.8. For IR (D–F), the calcium score was 59.9, and the image noise was 6.8. For DLIR (G–I), the calcium score was 59.1, and the image noise was 6.0. FBP, filtered back projection; IR, iterative reconstruction; DLIR, deep learning-based image reconstruction.

Attenuation within the chamber was 2.4 ± 1.1 with FBP, 2.7 ± 0.6 with IR, and 2.3 ± 0.9 with DLIR. Image noise within the chamber was 10.8 ± 0.3 with FBP, 6.8 ± 0.2 with IR, and 6.0 ± 0.1 with DLIR. Calcium scores were 59.3 ± 2.2, 59.9 ± 2.3, and 59.1 ± 1.9 with FBP, IR, and DLIR, respectively. Because these repeated measurements were obtained from a single calcium insert configuration, the phantom findings were interpreted descriptively, with measurement variability reported for each reconstruction method rather than subjected to formal statistical comparison. Fig 2 shows images reconstructed with FBP, IR, and DLIR.

### Clinical study

#### Demographic characteristics.

The patient characteristics and cardiovascular risk factors are summarized in Table 2. A total of 113 patients (69 men [61.1%] and 44 women [38.9%]) were enrolled in the study. The mean age of the patients was 66.9 ± 13.3 years, and the mean heart rate was 77.3 ± 16.1 beats per minute. Among the study population, 68 patients (60.2%) had hypertension, 38 (33.6%) had diabetes mellitus, 17 (15.0%) had dyslipidemia, and 23 (18.2%) were smokers. The mean CTDI_vol_ was 7.8 ± 6.7 mGy. The mean DLP was 126.0 ± 107.7 mGy·cm.

**Table 2:** Patient characteristics and cardiovascular risk factors.

Variables	Value
Age, years	66.9±13.3
Sex, n (%)	
Female	44 (38.9)
Male	69 (61.1)
Height, cm	164.5±9.4
Weight, kg	65.5±12.5
BMI, kg/m^2^	24.1±3.5
Heart rate, beats per minute	77.3±16.1
Dyslipidemia, n (%)	
No	96 (85.0)
Yes	17 (15.0)
Hypertension, n (%)	
No	45 (39.8)
Yes	68 (60.2)
Diabetes, n (%)	
No	75 (66.4)
Yes	38 (33.6)
Smoking status, n (%)	
No	90 (81.8)
Yes	23 (18.2)
CTDI_vol_ (mGy)	7.8±6.7
DLP (mGy·cm)	126.0±107.7

*BMI*, body mass index; *CTDI*, Computed Tomography Dose Index; *DLP*, dose–length product

### Image attenuation and noise

Image attenuation and noise were compared using parametric analyses. The attenuation was 45.3 ± 7.6 for FBP, 45.1 ± 8.2 for IR, and 45.4 ± 7.9 for DLIR. Noise was 20.6 ± 2.6 for FBP, 11.7 ± 1.9 for IR, and 10.1 ± 1.8 for DLIR. Post-hoc results for attenuation and noise are summarized in Table 3.

**Table 3:** Attenuation and image noise across FBP, IR and DLIR, using Repeated Measures ANOVA with post-hoc pairwise comparisons.

	mean±SD			post hoc analysis (mean difference±SD)					
	FBP	IR	DLIR	FBP vs IR	p-value	FBP vs DLIR	p-value	IR vs DLIR	p-value
Attenuation	45.3 ± 7.6	45.1 ± 8.2	45.4 ± 7.9	0.2 ± 3.2	0.589	−0.1 ± 3.3	0.714	−0.3 ± 2.6	0.259
Noise	20.6 ± 2.6	11.7 ± 1.9	10.1 ± 1.8	8.9 ± 1.4	<.0001	10.5 ± 2.4	<.0001	1.6 ± 1.6	<.0001

*FBP*, filtered back projection; *IR*, iterative reconstruction; *DLIR*, deep learning-based image reconstruction; *SD*, standard deviation; *IQR*, interquartile range

### Total CACS

Total CACS was analyzed using parametric and nonparametric methods to account for its zero-inflated distribution. The mean total CACS was 461.9 ± 959.3 for FBP, 450.9 ± 942.6 for IR, and 447.3 ± 935.8 for DLIR, with post hoc pairwise differences summarized in Table 4A. The median [IQR] of total CACS was 58.4 [0.8–326.0] for FBP, 53.0 [0.8–322.0] for IR, and 49.3 [0.0–322.0] for DLIR. CACS with DLIR was significantly lower than that with FBP (p < 0.001). Detailed differences were assessed using the Friedman test, and post hoc pairwise comparisons are summarized in Table 4B.

**Table 4:** Parametric and non-parametric analyses of CACS across FBP, IR, and DLIR.

(A) Repeated Measures ANOVA for the CACSs of the coronary arteries									
	mean±SD			post hoc analysis (mean difference±SD)					
	FBP	IR	DLIR	FBP vs IR	p-value	FBP vs DLIR	p-value	IR vs DLIR	p-value
LM	29.2±78.4	28.6±76.7	29.0±78.1	0.6±3.3	0.055	0.2±2.8	0.442	-0.4±3.6	0.239
LAD	176.2±335.8	171.2±328.2	172.0±329.8	5.0±12.9	<.0001	4.1±14.7	0.004	-0.9±8.4	0.266
LCx	82.9±233.6	82.3±233.6	81.0±231.2	0.6±9.5	0.476	1.9±7.0	0.005	1.2±10.3	0.205
RCA	173.4±418.4	168.8±411.0	165.7±403.7	4.5±11.9	<.0001	7.6±22.8	0.001	3.1±16.7	0.050
Total	461.9±959.3	450.9±942.6	447.3±935.8	11.0±23.4	<.0001	14.5±34.0	<.0001	3.5±23.9	0.120
**(B) Friedman test for the CACSs of the coronary arteries**									
	**Median [IQR]**			**post hoc analysis (median difference[IQR])**					
	**FBP**	**IR**	**DLIR**	**FBP vs IR**	**p-value**	**FBP vs DLIR**	**p-value**	**IR vs DLIR**	**p-value**
LM	0 (0.0-7.95)	0 (0.0-8.0)	0 (0.0-8.7)	0 (0.0-0.0)	<.0001	0 (0.0-0.0)	0.008	0 (0.0-0.0)	0.739
LAD	12.1 (0.0-198.0)	10.9 (0.0-190.0)	9.7 (0.0-170.0)	0 (0.0-4.0)	<.0001	0 (0.0-3.0)	<.0001	0 (-0.8-0.4)	0.353
LCx	0 (0.0-28.4)	0 (0.0-26.2)	0 (0.0-26.6)	0 (0.0-1.2)	<.0001	0 (0.0-1.4)	<.0001	0 (0.0-0.0)	0.115
RCA	0.8 (0.0-52.8)	0 (0.0-45.7)	0.8 (0.0-46.3)	0 (0.0-2.0)	<.0001	0 (0.0-3.2)	<.0001	0 (0.0-0.8)	0.004
Total	58.4 (0.8-326.0)	53 (0.8-322.0)	49.3 (0.0-322.0)	1.4 (0.0-10.5)	<.0001	2 (0.0-11.0)	<.0001	0 (-0.2-2.0)	0.064

*FBP*, filtered back projection; *IR*, iterative reconstruction; *DLIR*, deep learning-based image reconstruction; *LM*, left main artery; *LAD*, left anterior descending artery; *LCx*, left circumflex artery; *RCA*, right coronary artery; *CACS*, coronary artery calcium score; *SD*, standard deviation; *IQR*, interquartile range

### Correlation

Correlation coefficients between image quality and CACS were assessed across three sets. The correlation coefficient for attenuation between FBP and IR was 0.922, between FBP and DLIR was 0.912, and between IR and DLIR was 0.950. The correlation coefficient for noise between FBP and IR was 0.845, between FBP and DLIR was 0.456, and between IR and DLIR was 0.597. Pearson correlation coefficients for total CACS were near-perfect across all pairwise comparisons, as shown in Table 5 A. However, these values were interpreted only as supplementary descriptive measures because they reflect preserved rank ordering across reconstruction methods generated from the same raw acquisition data and do not establish clinical interchangeability. The correlation coefficients for the CACS of the individual coronary arteries are shown in Table 5 A.

**Table 5:** Correlation coefficient and ICC for the total and individual CACS from FBP, IR and DLIR.

(A) Correlation coefficients for image quality and the CACSs of the coronary arteries						
	Pearson’s correlation					
	FBP vs. IR		FBP vs. DLIR		IR vs. DLIR	
	r (95% CI)	p-value	r (95% CI)	p-value	r (95% CI)	p-value
LM	0.999(0.999-1.000)	<.0001	0.999(0.999-1.000)	<.0001	0.999(0.999-0.999)	<.0001
LAD	1.000(0.999-1.000)	<.0001	0.999(0.999-0.999)	<.0001	1.000(1.000-1.000)	<.0001
LCx	0.999(0.999-0.999)	<.0001	1.000(0.999-1.000)	<.0001	0.999(0.999-0.999)	<.0001
RCA	1.000(1.000-1.000)	<.0001	0.999(0.999-0.999)	<.0001	0.999(0.999-1.000)	<.0001
Total	1.000(1.000-1.000)	<.0001	1.000(1.000-1.000)	<.0001	1.000(1.000-1.000)	<.0001
(B) ICC for image quality and the CACSs of the coronary arteries						
	**ICC**					
	**FBP vs. IR**		**FBP vs. DLIR**		**IR vs. DLIR**	
	**ICC (95% CI)**	**p-value**	**ICC (95% CI)**	**p-value**	**ICC (95% CI)**	**p-value**
LM	0.9991(0.9986-0.9994)	<.0001	0.9994(0.9991-0.9996)	<.0001	0.9989(0.9984-0.9993)	<.0001
LAD	0.9991(0.9985-0.9995)	<.0001	0.9990(0.9984-0.9993)	<.0001	0.9997(0.9995-0.9998)	<.0001
LCx	0.9992(0.9988-0.9994)	<.0001	0.9995(0.9993-0.9997)	<.0001	0.9990(0.9986-0.9993)	<.0001
RCA	0.9995(0.9992-0.9997)	<.0001	0.9983(0.9973-0.9989)	<.0001	0.9991(0.9987-0.9994)	<.0001
Total	0.9996(0.9993-0.9998)	<.0001	0.9992(0.9986-0.9995)	<.0001	0.9997(0.9995-0.9998)	<.0001

Pearson correlation coefficients are presented to three decimal places. ICC values are presented to four decimal places to reduce rounding-related ambiguity. *FBP*, filtered back projection; *IR*, iterative reconstruction; *DLIR*, deep learning-based image reconstruction; *CACS*, coronary artery calcium score; *CI*, confidence interval; *LM*, left main artery; *LAD*, left anterior descending artery; *LCx*, left circumflex artery; *RCA*, right coronary artery.

Subsequently, the ICCs for the three sets of image quality and CACS measurements were analysed. The ICC for attenuation between FBP and IR was 0.919, between FBP and DLIR was 0.912, and between IR and DLIR was 0.949. The ICC for noise between FBP and IR was 0.092, between FBP and DLIR was 0.035, and between IR and DLIR was 0.436. This near-zero ICC for noise between FBP and DLIR is expected and does not indicate a failure of agreement, because FBP and DLIR are designed to produce systematically different noise levels. In this context, the low ICC reflects intentional differences in noise suppression rather than inconsistency in reconstruction performance. The ICCs for total CACS were 0.9996 between FBP and IR, 0.9992 between FBP and DLIR, and 0.9997 between IR and DLIR. Although these ICC values were very high, they were interpreted cautiously because near-perfect ICCs do not exclude individual-level disagreement or clinically relevant category reclassification. The near-perfect Pearson correlation coefficients for total CACS reflect the fact that FBP, IR, and DLIR were reconstructed from the same raw acquisition data in each patient, thereby preserving the rank ordering of scores across methods. Likewise, ICC values reported as 1.000 should be interpreted with caution, as these values reflect rounding of unrounded estimates close to, but not exactly, 1.000. The ICCs for the CACS of individual coronary arteries are shown in Table 5 B. Therefore, individual-level agreement was evaluated primarily using Bland–Altman analysis, CAC-DRS reclassification, and false-negative classification rather than Pearson correlation coefficients or ICCs alone.

### Bland–Altman analyses

**Fig 3:**
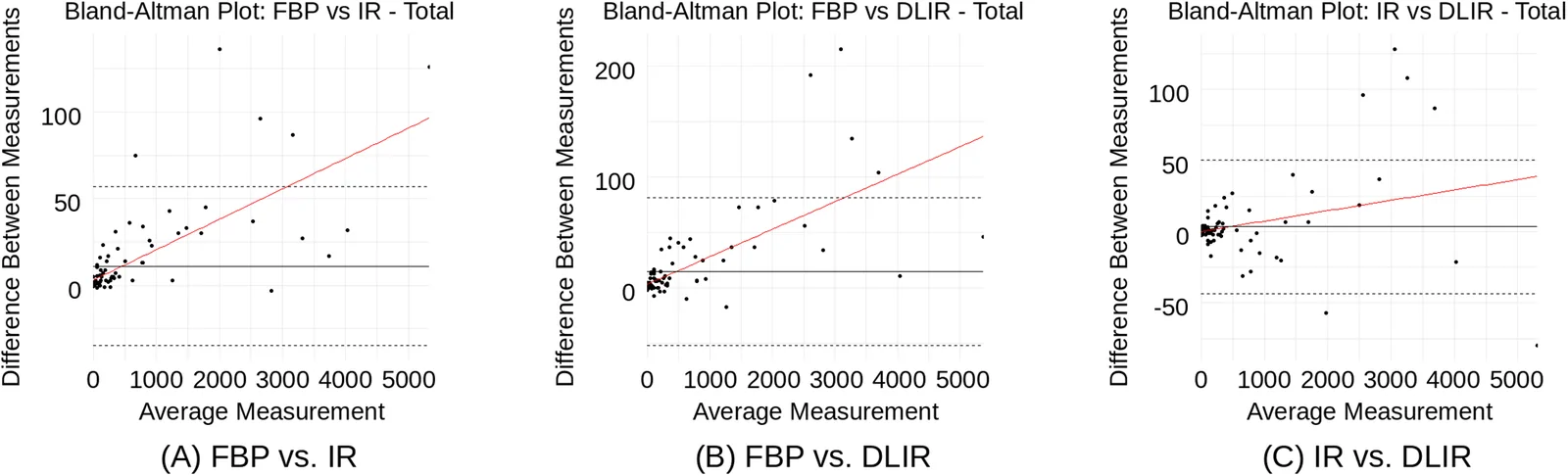
Bland–Altman plots for total CACS across FBP, IR, and DLIR. (A) FBP vs. IR, (B) FBP vs. DLIR, (C) IR vs. DLIR. The solid line indicates the mean difference, and the dashed lines indicate the 95% limits of agreement.

Three sets of Bland–Altman plots showing the mean difference and 95% limits of agreement (LoA) for image quality and total CACS are shown in Fig 3. The mean difference in total CACS between FBP and IR was 11.01, with a 95% LoA of –34.8 to 56.8. The mean difference in total CACS between FBP and DLIR was 14.5, with a 95% LoA of −52.1 to 81.1. The mean difference in total CACS between IR and DLIR was 3.5, with a 95% LoA of −43.3 to 50.3. The wide limits of agreement between FBP and DLIR suggest that, despite high overall concordance, clinically meaningful differences in total CACS may occur at the individual-patient level, particularly near CAC-DRS category boundaries. These findings indicate that near-perfect Pearson correlation coefficients and ICCs did not preclude clinically meaningful individual-level disagreement between reconstruction methods.

### CACS classification concordance

**Fig 4:**
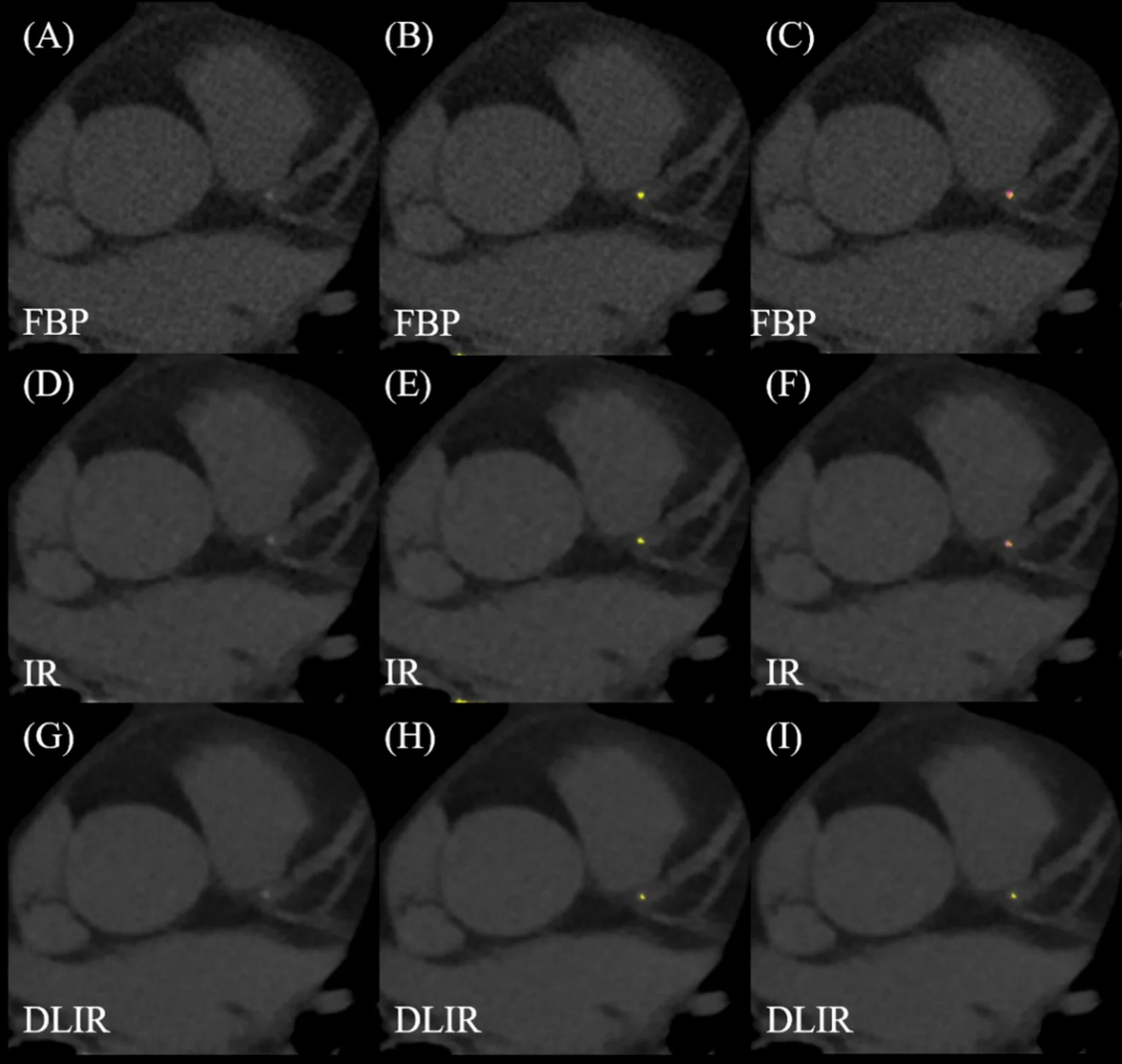
Comparison of CACS values in FBP, IR, and DLIR. All images represent original, de-identified clinical CT scans obtained at our institution in accordance with an IRB-approved study protocol, and do not reproduce any material from previously published works or external sources. Comparison of FBP, IR, and DLIR for detecting low calcium levels. The leftmost column shows standard CT images commonly used in clinical settings. The central column displays software-processed results, with calcium automatically detected and highlighted in yellow for values exceeding 130 HU. In the rightmost column, regions measuring ≥1 mm³ were subsequently validated as calcium by the reader and marked in pink to indicate manual confirmation. A–C: CCTA images reconstructed with FBP show CAC in the LAD, yielding a CACS of 1.17. D–F: The CACS remained stable at 1.17 with IR, indicating consistency between the two reconstruction methods. G–I: However, DLIR produced a false-negative result, yielding a CACS of 0 despite CAC in the LAD. This suggests that, compared with FBP and IR, DLIR may underestimate or overlook small calcifications. CACS, coronary artery calcium score; CCTA, coronary computed tomography angiography; CAC, coronary artery calcium; DLIR, deep learning image reconstruction; FBP, filtered back projection; IR, iterative reconstruction; LAD, left anterior descending artery.

CAC was categorized according to the CAC-DRS. CAC-DRS categories are as follows: 0 = very low risk; 1–99 = mildly increased; 100–299 = moderately increased; and ≥300 = moderate to severe increase [20–22]. Weighted kappa coefficients were 0.988 (95% confidence interval: 0.965–1.000) between FBP and IR, 0.952 (95% confidence interval: 0.906–0.998) between FBP and DLIR, and 0.964 (95% confidence interval: 0.924–1.000) between IR and DLIR. Among the same 113 patients, the distribution across CAC-DRS categories varied slightly according to reconstruction method. Using FBP, 27 patients were classified as CACS 0, 35 as <100, 21 as 100–299, and 30 as ≥300. With IR, 28 patients were classified as 0, 34 as <100, 21 as 100–299, and 30 as ≥300. For DLIR, the counts were 30 for CACS 0, 33 for <100, 20 for 100–299, and 30 for ≥300. Table 6 A shows that 112 of 113 patients were classified into the same CAC-DRS category by both FBP and IR. One patient was underclassified by IR despite a CACS > 0 on FBP, yielding a false-negative rate of 1.16% (1 of 86). No patients with a CACS of 0 were misclassified as having calcium, yielding a false-positive rate of 0.0% (0 of 27). Table 6 B shows that 109 of 113 patients were consistently classified between FBP and DLIR. DLIR underclassified four patients, yielding a false-negative rate of 4.65% (4 of 86). As with IR, the false-positive rate remained 0.0% (0 of 27), since no patient with a CACS of 0 was incorrectly placed in a higher category. Similarly, Table 6 C shows that 110 of 113 patients were consistently classified between IR and DLIR. DLIR underclassified three patients, yielding a false-negative rate of 3.53% (3 of 85). No patients with a CACS of 0 were misclassified as having calcium, yielding a false-positive rate of 0.0% (0 of 28) (Fig 4). A more detailed review of the downward-reclassified cases showed that these events occurred in patients with low calcium burden, where small differences in lesion attenuation or extent were sufficient to alter CAC-DRS categorization. Specifically, in three cases, calcium identified on FBP was completely undetected by DLIR, with FBP CACS values of 0.795, 0.795, and 1.19; all three were reclassified from CAC-DRS category 1 to category 0. These cases were considered clinically relevant because reclassification from a non-zero CACS to CACS 0 may alter risk interpretation and preventive treatment consideration in appropriate clinical contexts. In the remaining case, DLIR reduced the CACS from 110 on FBP to 95.2, resulting in reclassification from CAC-DRS category 2 to category 1. Bowker’s test of symmetry showed no significant asymmetry in the distribution of reclassified patients across methods (FBP vs. IR, p = 0.986; FBP vs. DLIR, p = 0.677; IR vs. DLIR, p = 0.809).

**Table 6:** Frequency tables for CACS classifications according to CAC-DRS risk categories.

(A) FBP vs. IR: Weighted Kappa 0.988 (95% CI: 0.965-1.000)					
	**IR**	**0**	**Total < 100**	**100 ≤ Total < 300**	**Total ≥ 300**
**FBP**					
**0**		27	0	0	0
**Total < 100**		1	34	0	0
**100 ≤ Total < 300**		0	0	21	0
**Total ≥ 300**		0	0	0	30
(B) FBP vs. DLIR: Weighted Kappa 0.952 (95% CI: 0.906-0.998)					
	**DLIR**	**0**	**Total < 100**	**100 ≤ Total < 300**	**Total ≥ 300**
**FBP**					
**0**		27	0	0	0
**Total < 100**		3	32	0	0
**100 ≤ Total < 300**		0	1	20	0
**Total ≥ 300**		0	0	0	30
(C) IR vs DLIR: Weighted Kappa 0.964 (95% CI: 0.924-1.000)					
	**DLIR**	**0**	**Total < 100**	**100 ≤ Total < 300**	**Total ≥ 300**
**IR**					
**0**		28	0	0	0
**Total < 100**		2	32	0	0
**100 ≤ Total < 300**		0	1	20	0
**Total ≥ 300**		0	0	0	30

*FBP*, filtered back projection; *IR*, iterative reconstruction; *DLIR*, deep learning-based image reconstruction; *CACS*, coronary artery calcium score; *CAC-DRS*, Coronary Artery Calcium Data and Reporting System

### Inter-observer agreement

Calcium scores were independently assessed by two experienced readers in 34 patients (30% of 113 participants), and inter-observer agreement was evaluated using the ICC. Each reader independently performed the full semiautomated calcium-scoring workflow across all three reconstruction datasets. Aquarius software automatically highlighted regions exceeding 130 HU, and each reader independently reviewed these candidate regions and confirmed or excluded them as calcifications. Because this workflow was based on a fixed threshold, reader-dependent variability was negligible, resulting in inter-observer ICC values of >0.999 for FBP, IR, and DLIR. No formal disagreement resolution process was required.

## Discussion

This study showed that DLIR achieved high overall concordance with FBP and IR for coronary calcium scoring while reducing image noise. However, DLIR showed systematic underestimation and non-negligible individual-level disagreement from FBP, with false-negative CACS 0 reclassification in a small subset of patients. These findings indicate that DLIR-derived CACS should not be regarded as fully interchangeable with FBP-derived CACS.

In general, the CACS is measured using images reconstructed with FBP, the primary method when the Agatston score was initially developed [5]. However, advances in computing technology have led to newer reconstruction techniques, prompting studies comparing CACSs obtained with different methods [23,24]. Among these, IR has been widely adopted in clinical practice owing to its ability to reduce noise while preserving anatomical information [25]. This technique reduces noise by iteratively refining the image data until predefined convergence criteria are met [26]. Recently, as AI has garnered attention, there has been considerable interest in biomedical images trained with deep neural networks [27]. This enables acquisition of the desired coronary artery images at a lower dose than with IR [13,28]. Benz et al. [29] reported that DLIR reduces radiation dose by 43% compared with coronary CT angiography, without significantly affecting image noise, stenosis severity, plaque composition, or quantitative plaque volume. Despite these advantages, the effects of DLIR on calcium scoring have not yet been widely evaluated. Our phantom study showed that the mean CACS across the three reconstruction methods remained within a narrow range (59–60) under standardized conditions. However, this result should be interpreted only as a limited technical assessment, because the phantom experiment used a single calcium insert configuration and did not include variable calcification sizes, densities, or morphologies. Therefore, the phantom findings cannot fully explain the false-negative findings observed in the clinical cohort, particularly for small or low-density calcifications near the 130-HU threshold. Similar to our findings, Dobrolinska et al. [30] also showed that both IR and DLIR can be used as alternatives to FBP in CACS. Recent studies have also reported lower Agatston scores with DLIR, particularly at higher strength levels [17].

In clinical applications, new imaging techniques need to be validated. Our findings indicate that although the correlation and concordance of CACS between FBP and DLIR were high, a false-negative rate of 4.65% was still observed. More importantly than the near-perfect Pearson correlation coefficients and ICCs, the Bland–Altman analysis showed wide limits of agreement between FBP and DLIR. In particular, the mean difference between FBP and DLIR was 14.5, with 95% limits of agreement from −52.1 to 81.1. Although the average difference was modest, the width of the limits of agreement indicates that DLIR-derived scores may differ meaningfully from FBP-derived scores in individual patients. This is particularly relevant near CAC risk thresholds such as 0, 100, and 300, where relatively small absolute changes in Agatston score may alter risk-category assignment and clinical interpretation. Such discrepancies may result in score-based reclassification and could influence clinical interpretation, preventive treatment decisions, and patient risk communication in low-burden cases. A false-negative result that reclassifies a patient from a non-zero CACS to a score of zero is clinically important, because a CACS of zero is interpreted as indicating very low cardiovascular risk and may influence patient risk communication and preventive treatment decisions, including statin consideration in appropriate clinical contexts. Rossi et al. [15] found that although the Agatston score was underestimated, the DLIR method reduced noise, thereby enhancing image quality. The presence of false negatives, although limited, underscores the need for careful interpretation when applying DLIR to calcium scoring [15,30]. One possible explanation is that high-strength GE TrueFidelity DLIR may affect calcium scoring through noise suppression and image smoothing. Because Agatston scoring depends on a fixed 130-HU threshold, even a small reduction in peak attenuation, edge sharpness, or apparent lesion extent may reduce the number of above-threshold voxels in very small, low-density, or partially volume-averaged calcifications. This may lower the measured calcium score or convert a small positive CACS to zero in very low-burden cases. However, this mechanism was not directly proven in the present study, because the phantom experiment used a single calcium insert configuration and did not include systematically varied calcification sizes, densities, or morphologies. Although our results suggest that DLIR has the potential to improve image quality, radiation dose was identical across reconstruction methods in this study because all three methods were applied to the same raw acquisition data; therefore, dose reduction was not directly evaluated. This benefit must be weighed against the risk of underestimating the calcium burden, which could influence clinical decision-making. In particular, the potential for underestimation must be considered in clinical environments [31], where precise calcium scoring is essential. One important consideration is that calcium scores may appear slightly reduced during follow-up, depending on the reconstruction method applied. Follow-up evaluations in clinical practice are frequently conducted to assess variations in CAC scores. Performing an initial scan with FBP, followed by a subsequent scan with DLIR, may be misinterpreted as a decrease in the calcium burden, potentially leading to an inaccurate assessment of disease progression. Furthermore, discrepancies in follow-up assessments may affect clinical decision-making, especially regarding the intensification of preventive measures or the reclassification of cardiovascular risk. Clinicians must ensure that changes in CACSs accurately represent disease progression rather than technical variations. They must recognize that alterations in the reconstruction methodology may affect CACSs and should consider this when interpreting follow-up studies. These findings also highlight the need for appropriate training and education for radiologists, technologists, and clinicians regarding reconstruction-specific effects, careful interpretation of DLIR-derived CAC 0 results, and consistent reconstruction protocols in follow-up examinations.

Furthermore, new methods should be carefully evaluated against established criteria rather than embraced [32,33]. Although DLIR shows promise, our study emphasizes that further validation through larger cohort studies is required to demonstrate its dependability. From a clinical implementation perspective, the present findings should be interpreted with caution because the evaluated DLIR algorithm was vendor-specific and may not perform identically across heterogeneous scanner platforms. Given that the internal architecture, training data, and reconstruction process of proprietary DLIR are not accessible to users, reproducibility should be supported through transparent protocol reporting and external multicenter and multivendor validation. In addition, the clinical impact of DLIR-related underestimation may vary across patient populations, baseline cardiovascular risk profiles, screening practices, and healthcare environments, limiting direct extrapolation from a single Korean tertiary-center cohort. Although the use of commercially available, regulatory-approved reconstruction software mitigates some regulatory concerns, broader clinical adoption will require further validation in multi-center, multi-vendor, and prospective settings. Given the increasing reliance on AI-based reconstruction techniques in medical imaging, ongoing assessment and standardization efforts are crucial to ensure optimal patient outcomes [34].

This study has several limitations. First, this was a single-center study with a modest sample size of 113 patients and no formal power analysis; the findings should be interpreted as feasibility and clinical risk-characterization results rather than definitive validation. Because only four false-negative events were observed, formal subgroup analysis, threshold sensitivity evaluation, or decision-impact modelling was not performed to avoid statistically unstable interpretation. In addition, because the evaluated method was a vendor-specific DLIR implementation (GE TrueFidelity at High strength), the results may not be directly applicable to algorithms from other vendors or scanner platforms. Second, the retrospective design may have introduced selection bias. Although major cardiovascular risk factors were recorded, standardized symptom data and other clinical parameters necessary for formal guideline-based risk or clinical likelihood assessment were not systematically obtained. Consequently, patients could not be reliably classified according to established cardiovascular risk or clinical likelihood categories, and how CACS reclassification translates across different risk profiles remains unclear [35]. Before DLIR is implemented in routine clinical practice, prospective validation incorporating standardized cardiovascular risk and symptom assessment is required. Third, the phantom study was limited to a single calcium insert configuration, which restricts generalizability to the broader range of coronary calcifications encountered in clinical practice. In particular, this design could not fully characterize DLIR behavior for small, low-density, or irregular calcifications, which may be more susceptible to underestimation or false-negative classification. Therefore, the phantom experiment should be interpreted as a limited standardized technical assessment rather than as comprehensive validation of DLIR for CAC scoring. Systematically varied calcification sizes, densities, and morphologies are needed to better define the reliability of DLIR for calcium scoring. Fourth, in this study, CAC scoring was performed by a limited number of readers using a single software program. Future prospective studies should use randomized, blinded, and independently adjudicated image-review workflows to further reduce potential observer bias. To validate our results and develop standardized processes for the application of DLIR in CAC evaluation, further studies with larger sample sizes and more diverse reconstruction settings are required.

## Conclusions

CACS with DLIR showed high overall concordance with CACS with FBP and IR. However, a degree of systematic underestimation was observed, and caution is warranted when interpreting these results, particularly in patients with low calcium burden or scores near CAC risk-category thresholds.

## Abbreviations

CACS: coronary artery calcium score
CAC-DRS: Coronary Artery Calcium Data and Reporting System
CCTA: coronary computed tomography angiography
DLIR: deep learning-based image reconstruction
FBP: filtered back projection
IHD: ischemic heart disease
IR: iterative reconstruction
HU: Hounsfield unit
ROI: region of interest
IQR: interquartile range
